# Game-Theoretic Methods for Functional Response and Optimal Foraging Behavior

**DOI:** 10.1371/journal.pone.0088773

**Published:** 2014-02-28

**Authors:** Ross Cressman, Vlastimil Křivan, Joel S. Brown, József Garay

**Affiliations:** 1 Department of Mathematics, Wilfrid Laurier University, Waterloo, Ontario, Canada; 2 Institute of Entomology, Biology Centre, Academy of Sciences of the Czech Republic, and Faculty of Science, University of South Bohemia, České Budějovice, Czech Republic; 3 Department of Biological Sciences, University of Illinois at Chicago, Chicago, United States of America; 4 MTA-ELTE Theoretical Biology and Evolutionary Ecology Research Group and Department of Plant Systematics, Ecology and Theoretical Biology, Eötvös Loránd University, Budapest, Hungary; Vrije Universiteit, Netherlands

## Abstract

We develop a decision tree based game-theoretical approach for constructing functional responses in multi-prey/multi-patch environments and for finding the corresponding optimal foraging strategies. Decision trees provide a way to describe details of predator foraging behavior, based on the predator's sequence of choices at different decision points, that facilitates writing down the corresponding functional response. It is shown that the optimal foraging behavior that maximizes predator energy intake per unit time is a Nash equilibrium of the underlying optimal foraging game. We apply these game-theoretical methods to three scenarios: the classical diet choice model with two types of prey and sequential prey encounters, the diet choice model with simultaneous prey encounters, and a model in which the predator requires a positive recognition time to identify the type of prey encountered. For both diet choice models, it is shown that every Nash equilibrium yields optimal foraging behavior. Although suboptimal Nash equilibrium outcomes may exist when prey recognition time is included, only optimal foraging behavior is stable under evolutionary learning processes.

## Introduction

The functional response [1], [2] considers the number of prey (or resource items) consumed by a single predator (or forager) as influenced by prey abundance. By dictating the mortality rate of prey and the feeding rate of predators, it is central to understanding consumer-resource dynamics [3], [4]. Furthermore, the functional response can be extended to consider a predator seeking two prey types [1]. Besides being more realistic for many predators, functional responses on two food types create indirect effects between the prey via the shared predator. For instance, if consuming a prey item takes time or reduces motivation, then the presence of a second food type decreases the forager's consumption of the first food type. Via the functional response, such prey become indirect mutualists [5]. Conversely, short-term apparent competition [6], [7] results if the presence of the second prey encourages the predator to spend more time or effort searching for and capturing prey. This happens when foragers bias their efforts towards areas rich in resources. Regardless, the two-food functional response is central to understanding diets, optimal foraging for multiple resources, predator mediated indirect effects between prey, and population dynamics within food webs.

Two modeling approaches have addressed the question of diet choice for a forager that searches for and then handles encountered prey items. The first is found in classic optimal foraging models. The forager's encounter probability or attack rate [3] is viewed as a mass action phenomenon between the predator and its prey. The forager's overall encounter rate with prey is simply the product of prey abundance and the predator's encounter probability on that prey. Upon encountering a prey, the forager can elect to consume the prey at some handling time cost, or reject the opportunity and continue the search for other prey. Starting from Holling's [1] two-food functional response this approach has generated increasingly sophisticated predictions.

In Pulliam [8] (see also [9]), a “zero-one” or “bang-bang” rule for diet choice was derived. A forager should either always accept or always reject an encountered food item. When encountered, the preferred food (based on a higher reward to handling time ratio) should always be consumed. If searching for and handling the preferred food type yields more (or less) reward than simply handling the less preferred food, then the less preferred food should always be rejected (or accepted) when encountered. Empirical support was encouraging but equivocal [10]. Most foragers show a partial selectivity, they are neither completely opportunistic nor completely selective. A number of mechanisms have been proposed and modeled for why foragers sometimes only partially consume a less preferred food; including food depletion [11], food bulk and digestion limitations [12], complementary nutrients [13], local omniscience [14], incorrect prey classification and sampling by predators [15], [16], prey crypsis [17] etc.

A second approach to diet choice is emerging from spatially-explicit models such as agent based models. A forager may move through a lattice or some form of continuous space. Prey items may occur at fixed locations or may also move through the defined space. The forager possesses some detection radius. Upon detecting a prey, the forager can choose to ignore the prey or attempt a capture. Such approaches lead to greater realism by considering the roles of space and individual contingencies. While they move through the same landscape, each individual forager becomes more or less unique based on its own personal history of movement, food encounters, and foraging decisions. Some individuals may experience unusually high or low harvest rates as a consequence of runs of good or bad luck, respectively. Like the classical models of diet choice, the foragers can still make optimal foraging decisions by deciding which encountered foods to handle or reject. The simulations can be run with a myriad of decision rules, and the performance of these rules can be compared. While a best diet choice rule may emerge from a particular scenario, the explicit nature of the agent based models may obscure the elegance or simplicity of the decision rule. Such agent based models may approximate more or less the optimal decision rules from the first approach to diet choice [14].

Here we develop a decision theory approach to diet choice. We use an explicit decision tree to evaluate the costs and benefits of different choices. Such a decision tree has similarities to extensive form games from game theory [18], [19]. Our goals are threefold. First, does an explicit consideration of decision making recover the results from the classic “mass-action” models of diet choice. Second, can these decision trees assist in uncovering the optimal decision rules for agent-based foraging models. Third, what are the similarities and differences between the decision tree of a forager and evolutionary games in extensive form. To achieve these goals we imagine a forager that searches for and handles food items of two types.

We consider three different scenarios based on the nature of searching for food and the ability to recognize a food's type upon encounter. In the first, search is undirected in terms of food type, but upon encountering a food item the forager instantly recognizes its type. This accords with the assumptions that generate Holling's two-food functional response and an “all or nothing” decision rule of food type acceptability. In the second the forager may encounter one prey of each type (called simultaneous encounter [20]), but can only handle one of the items, the other being lost. For instance, these two prey may be together at the same place competing over a common resource. Alternatively, the predator may search a small area completely for any prey before deciding whether to attack. In the third, we consider recognition time where the forager must expend additional time if it wants to know the type of food that has been encountered prior to handling.

## Methods

### Decision trees and the functional response for two prey types

In this section, we develop a decision tree method to derive the predator's functional response. The tree details the predator-prey interactions under consideration. We envision several prey types spatially distributed among many patches (that we will call microhabitats). The encounter events are then partially determined by the prey through their spatial distribution before the predator arrives. For instance, if prey are territorial, then the predator can encounter at most one solitary prey in a given microhabitat. At another extreme, if the different types of prey aggregate, then the predator can encounter different prey types at the same time. Thus, encounter events depend on the spatial behavior of the prey.

We break the predation process into different stages. A typical predation process has at least three stages that answer the following questions: 1. What prey (or types of prey) does the predator encounter? 2. What does the predator do in a given encounter situation (e.g. does the predator attack a prey, what type does it attack, etc.)? 3. Is the predator successful or not if it attacks? Here, we construct functional responses from the underlying decision trees based on three scenarios. This construction is, however, quite general and described fully in section Decision trees and the functional responses of Appendix S1. We start with a well known example that leads to the Holling type II functional response for two prey types.

Suppose that there are two types of prey 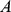 and 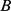 with fixed densities 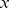 and 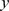, respectively. We assume that these prey are scattered randomly among 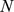 microhabitats where 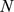 is much larger than the number of individuals (i.e., 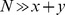). Thus, there will be at most one prey in each microhabitat (i.e. the probability that there are two or more in some microhabitat is negligible). Thus, the probabilities that a given microhabitat has no prey is 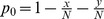, exactly one prey 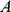 is 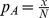 and exactly one prey 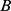 is 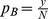. These probabilities are assumed not to change with time, which is the usual assumption when deriving a functional response.

**Figure 1:**
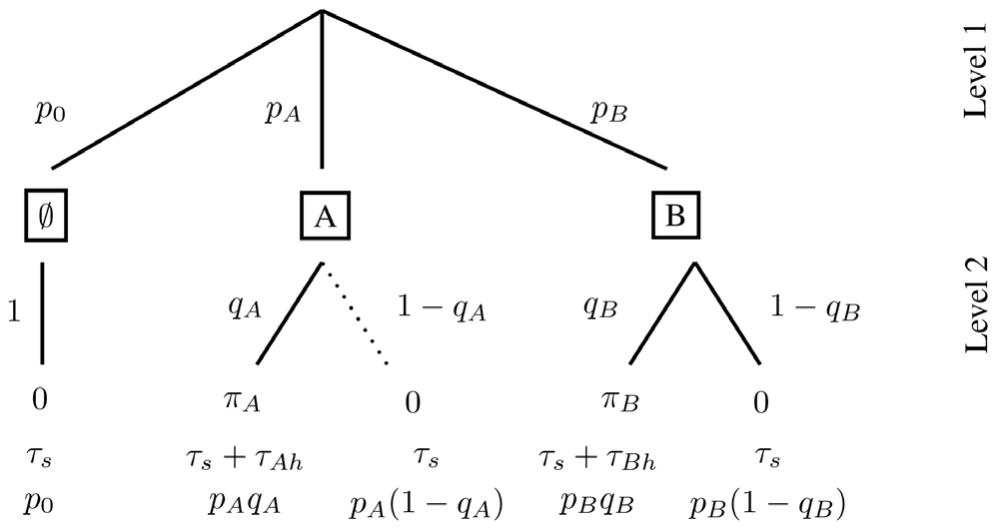
The decision tree for two prey types. The first level gives the prey encounter distribution. The second level gives the predator activity distribution. The final row of the diagram gives the probability of each predator activity event and so sum to 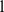. Since each entry here is simply the product of the probabilities along the path leading to this endpoint, we do not provide them in the decision trees from now on. With random prey distribution and 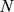 large, 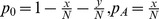 and 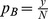. If prey 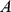 is the more profitable type, the edge in the decision tree corresponding to not attacking this type of prey is never followed at optimal foraging (indicated by the dotted edge in the tree). The reduced tree is then the resulting diagram with this edge removed.

Suppose the predator chooses a microhabitat to search at random, that it always finds the prey in this microhabitat if there is one, and that it takes a searching time 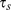 for it to determine whether a prey is there or not. There are then three possible encounter events: the predator encounters a prey of type 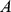, a prey of type 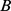, or no prey at all. These events occur with probabilities 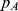, 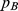 and 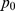 respectively (see Figure 1, Level 1).

For the first event when encountering prey 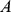, the predator has two possible actions: Either “attack prey 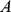
“ and “do not attack prey 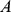.” These actions occur with probabilities 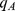 and 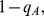 respectively (see Figure 1, Level 2). Similarly, in the second event when a predator encounters prey 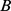, the two possible actions of the predator are to “attack prey 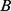
“ and “do not attack prey 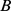
“ with probabilities 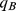 and 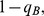 respectively. For the third event, when no prey are found, the only predator action is “do not attack” with probability 1. Altogether, there are five possible predator activities, and these correspond to the five edges at Level 2 in the decision tree of Figure 1.

Let the predator's handling times of prey 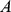 and 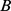 be 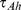 and 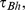 respectively. The five predator activity events are: encounter a microhabitat with prey 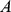 and attack it; encounter a microhabitat with prey 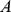 and do not attack it; encounter a microhabitat with prey 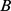 and attack it; encounter a microhabitat with prey 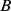 and do not attack it; encounter an empty habitat. The probability distribution of these activities (i.e. the “activity distribution”, [21]) in this order is 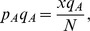 
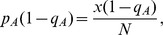
 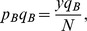 
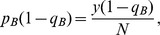
 
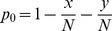
 with duration times 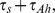 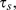 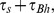 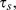 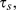 respectively. All this information is included in the decision tree of Figure 1. Also included in this tree are the energy consequences (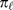) to the predator of each of the five activities.

Calculation of functional responses is based on renewal theory (for details, see section Decision trees and the functional responses of Appendix S1) which proves that the long term intake rate of a given prey type can be calculated as the mean energy intake during one renewal cycle divided by the mean duration of the renewal cycle [20], [22]-[24]. A single renewal cycle is given by a predator passing through the decision tree in Figure 1. Since type 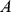 prey are only killed when the path denoted by 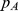 and then 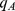 is followed, the functional response to prey 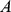, 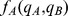, is given through Figure 1 by
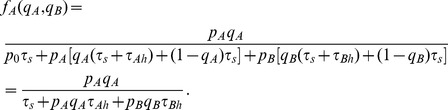



Similarly, the functional response for prey 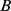 is
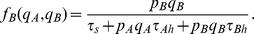



These are the functional responses assumed in standard two prey models (e.g., [9], [20], [25]) given in our notation. For instance, if we normalize searching time so that 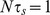, 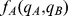 can be rewritten in terms of prey density in the more familiar form 
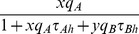
. As mentioned above, it is assumed that the encounter rates, 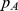 and 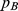, remain unchanged over the renewal cycle in that predation has negligible effect on prey densities during this time. This occurs if, for example, 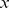 and 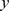 are large or 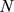 is quite large and so predation is rare. Our decision tree approach provides a mechanistic foundation to typical functional responses assumed in the literature. In particular, it is obvious that the standard Holling II functional response [2] given by 
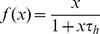
 is the outcome for Figure 1 when there is only one type of prey and the predator always pursues every prey it encounters (take 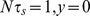 and 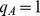).

The predator's rate of energy gain, 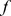, is given by (Figure 1)
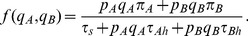
(1)


Like others [9], [20], [23], [26], we assume that the forager aims to maximize 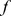. This theory predicts that if the two types of prey are ranked according to their “profitabilities” (i.e. their respective nutritional values per unit of handling time 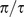), then the more profitable prey type is always included in the diet. That is, if 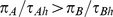, then the optimal foraging strategy is to attack all encountered prey 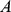 (i.e. 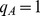). Furthermore, the decision to attack the lower ranked prey (i.e. prey B) satisfies the zero-one rule. Specifically, 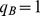 (respectively, 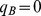) if its profitability is greater than (respectively, less than) the nutritional value of only attacking prey of type A (i.e. 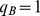 if and only if 
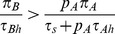
). The threshold value for including the less profitable prey in the predator's diet depends only on the chances of encountering the more profitable prey (i.e. only on the density of prey 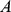) since 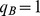 if and only if 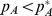 where
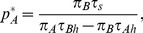
(2)
[9], [20], [23], [26].

### Decision trees and extensive form games

The decision tree approach is reminiscent of games given in extensive form [18], [19]. Because of this relationship between decision trees and extensive form games, game theory can then be used to find the optimal foraging strategy. First, we use the truncation method to eliminate those paths that always yield suboptimal outcomes. When applied to Figure 1, truncation removes the dotted path of rejecting the opportunity to capture prey type A. It is never optimal to reject the prey that offers a higher reward to handling time ratio. But what of node B? For food B with a lower energy to handling time ratio, we can find the optimal foraging strategy by analyzing the agent normal form [19]. This method assigns a separate player (called an agent) to each decision node. The possible decisions at this node become the agent's strategies and its payoff is given by the total energy intake rate of the predator it represents. When game theory is used to solve a single predator's decision tree, all of the virtual agents have the same common payoff, and in a sense, these agents engage in a cooperative game. The optimal foraging strategy of the single predator is then a solution to this game.

To illustrate the approach, we make the decision tree of Figure 1 into a two-player foraging game. Player 1 corresponds to decision node A with strategy set 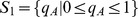 and player 2 to node B with strategy set 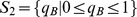. Their common payoff 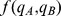 is given by (1). In an extensive form game, the payoff functions are linear in the behavioral strategy choices of all players. For our optimal foraging games, these payoffs are nonlinear functions and so are more similar to those found in population games [27], [28]. As a game, we seek the Nash equilibrium (NE). This is a pair of behavioral strategies 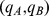, one for each player, such that neither player can gain by unilaterally changing its strategy. That is,
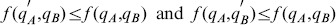
(3)for all 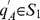 and 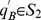. In game-theoretic terms, 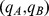 is a NE if 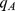 is a best response of player 1 to 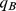 and 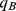 is a best response of player 2 to 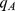.

**Figure 2:**
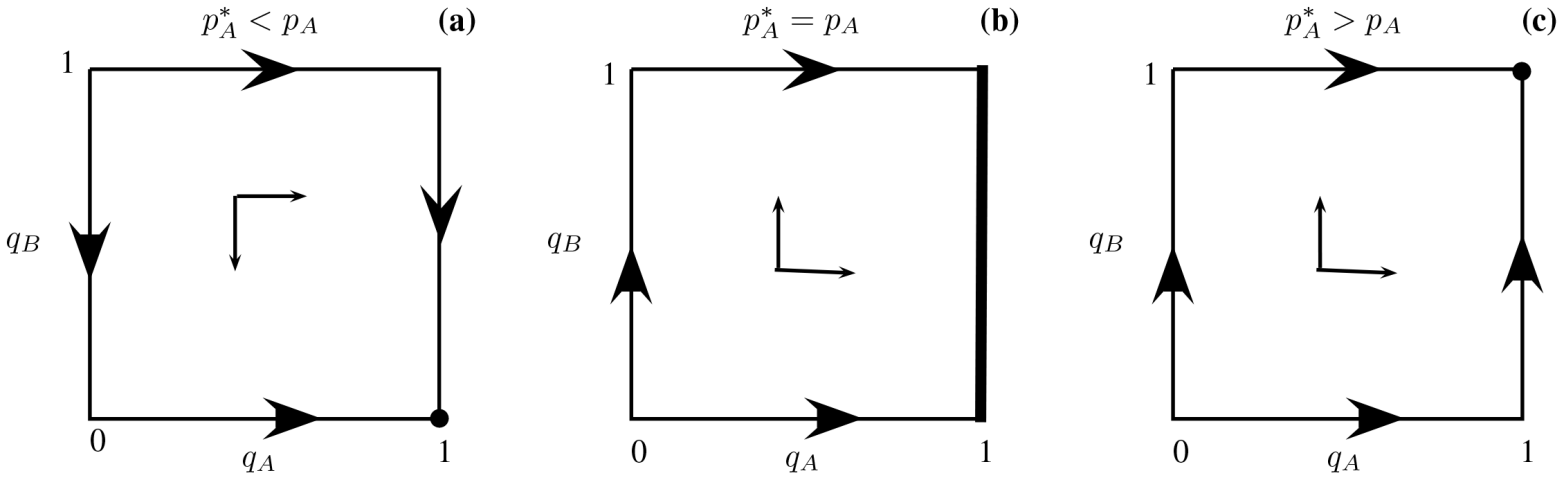
Qualitative outcomes of the optimal foraging strategy for the classical foraging model (1) with two prey types as a function of the encounter probability with the most profitable prey (i.e. of 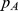). Panel (a) assumes that 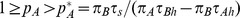 in which case the optimal strategy and NE is 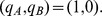 In panel (c), 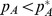 and the optimal strategy (and NE) is 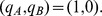 The arrows in each panel indicate the direction of increasing energy intake per unit time at points in the unit square. For completeness, the figure also includes the threshold case, panel (b), where 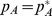 (i.e. the density of 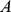 prey is at the switching threshold). Although this case is rarely considered by ecologists, its inclusion here is important to understand the optimal outcomes in our more complicated models. In panel (b), the optimal strategy is 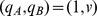 where 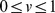, corresponding to the solid right-hand edge of the unit square that forms a set of NE points.

Clearly, an optimal foraging behavior 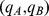 (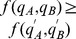 for all 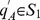 and 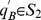) corresponds to a NE since it satisfies (3). Solving the game (i.e. finding the NE) for the classic foraging model of two types of prey is straightforward. Since 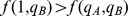 for all 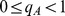 and 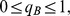 the behavioral strategy of player 1 to attack (i.e. 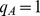) strictly dominates all its other options (i.e. 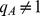) and so, at any NE, player 1 must play 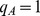. The NE strategy of player 2 is then any best response to 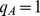 (i.e. any 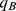 that satisfies 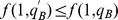 for all 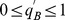). A short calculation yields
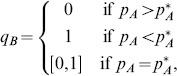
(4)where 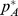 is given by (2). These results are shown in Figure 2 where NE are indicated by solid circles (panels (a) and (c)) and by the solid line segment on the right edge of panel (b). In this latter case (i.e. when 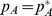), every point on this vertical edge 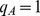 is a NE and the entire edge forms a NE component (i.e. a maximal connected set of NE, cf. [19]). Thus, at this critical encounter rate with the more profitable prey type, the zero-one rule of optimal foraging which states that a given resource type in a given patch is either always consumed when encountered or never consumed, must be modified because the optimally foraging predator preference for the alternative prey type can be anywhere between 0 and 1.

Since Figure 1 is a two-level foraging game, Theorem 3 of section Zero-one rule and the Nash equilibrium of Appendix S1 implies that the NE given by Figure 2 (i.e. by 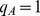 and 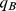 given by (4)) completely characterize optimal predator foraging behavior. Figure 2 also indicates the direction of increasing energy intake per unit time at points in the unit square. This suggests yet another connection to game theory; namely, how does the predator learn its optimal behavior? This question is commonly studied in evolutionary game theory [19], [29] where individual behaviors evolve in such a way that strategies with higher payoff become used more frequently. By following the flow of increasing payoff in the figure, it is clear from Figure 2 that such an evolutionary process will automatically lead to optimal predator behavior. We will return to this question in section Game theory and evolutionary outcomes for the prey recognition game where the evolutionary outcome is not so clear.

In these more general games where the decision tree has more than 2 levels, there may be NE that do not correspond to optimal foraging behavior. However, so long as the number of encounter events at level 1 and predator activities remain finite, these decision trees generate the predator's energy intake rate and its functional responses on each type of prey. Game-theoretic equilibrium selection techniques [30] based on evolutionary outcomes can then be used to discard suboptimal NE behaviors and select only those NE corresponding to optimal foraging behaviors as we will see in the final example that includes prey recognition effects (see section Prey Recognition Effects).

## Results

### Foraging with simultaneous resource encounters

**Figure 3:**
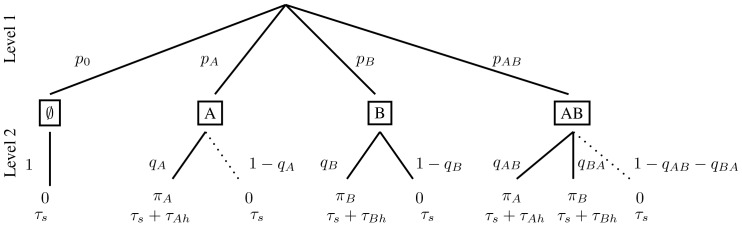
The decision tree for the simultaneous encounter game. At optimal foraging, two edges of this tree diagram are never followed. These are indicated by dotted lines in the tree. The reduced tree is then the resulting diagram with these edges removed.

In this section, we again assume that there are two resource types (denoted as 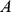 and 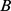) but, unlike section Decision trees and the functional response for two prey types, some microhabitats can contain a mixture of both types (denoted as 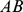). In this case, we assume that the consumer can forage for at most one resource type in any encounter event. Other microhabitats can be resources free. Furthermore, let 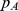, 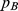 and 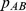 respectively be the proportions of these microhabitats that contain only resource 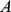, only resource 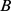 prey and both resources 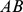 respectively. Finally, let 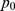 be the proportion of microhabitats that contain no resources. If the consumer chooses a patch at random, the distribution of encounter events is given by Level 1 of Figure 3.

Figure 3 also contains the distribution of consumer activity events under the assumption that the consumer is always successful when it decides to forage a resource that it encounters. In the predator-prey interpretation, this means the predator kills its prey whenever it attacks. As discussed in the final paragraph of section Decision trees and the functional responses of Appendix S1, our decision tree approach to optimal foraging is also applicable when the attacking predator is only successful with a certain probability that may depend on the type of prey. Here 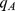 (respectively, 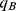) is the probability the consumer forages for the resource when it encounters only resource type 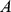 (respectively, type 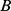). Also 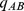 (respectively, 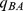) is the probability the consumer forages type 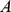 (respectively, type 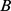) resource when it chooses a microhabitat that contains both types of resources and so 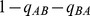 is the probability the consumer decides not to forage for either resource in this encounter event.

The functional response can then be developed from the decision tree in Figure 3 that includes the searching and handling times as well as the energy intakes of the different activity events. Proceeding as in section Decision trees and functional response for two prey types, the functional responses to resource type 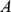 and 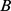 are given by
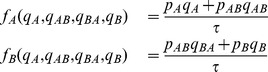
(5)respectively, where 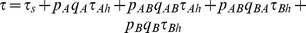. Thus the total consumer energy intake per unit time is
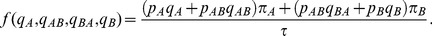
(6)


To find the optimal foraging strategy, we solve for the NE of the three-player game that assigns one player to each of the consumer decision nodes in Figure 3. As shown in section Foraging with simultaneous resource encounters of Appendix S1, the behavior strategy to consume resource 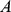 at node A strictly dominates all other actions of this player (i.e., 
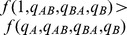
 for all 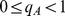), as we assume that resource 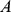 is more profitable to the predator than resource 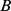 (i.e. that 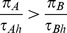). It is also shown there that any behavior strategy at node AB whereby a resource is not always consumed (i.e. 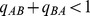) is strictly dominated. Thus 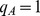 and 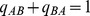 at any NE.

From these two results, the decision tree in Figure 3 can be truncated by deleting the two edges indicated by dotted lines. With this change, the consumer energy intake rate 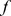 becomes
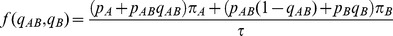
(7)where now 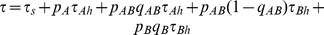.

Thus, the optimal strategy is a NE of the two-player game corresponding to the reduced tree of Figure 3. In this two-level foraging game, player 1 corresponds to decision node AB with strategy 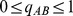 and player 2 at node B with strategy 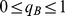. Their common payoff is given by (7). From section Foraging with simultaneous resource encounters of Appendix S1 the best response for player 1 that encounters both prey types simultaneously given the current strategy of player 2 is
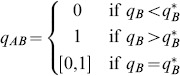
(8)where
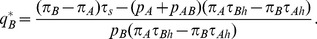
(9)


Similarly, the best response of player 2 when encountering only resource 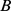 is
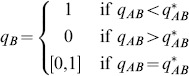
(10)where
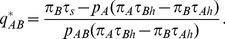
(11)


Then 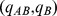 is a NE if and only if this strategy pair satisfies equations (8) and (10). Thus, unlike section Decision trees and functional response for two prey types, the NE behavior at one consumer decision node depends on the behavior at the other.

**Figure 4:**
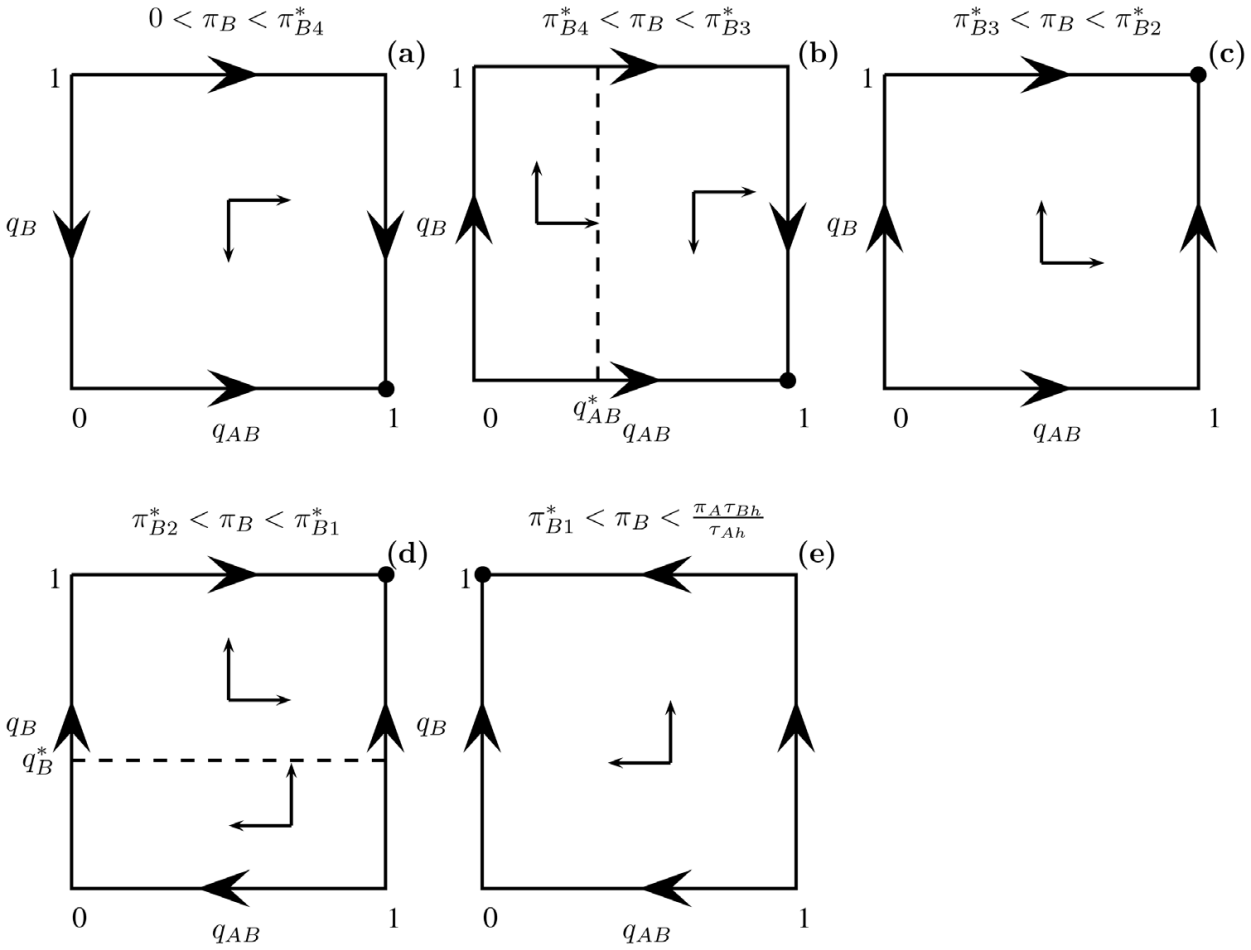
All qualitative outcomes of the optimal foraging strategy (8) and (10) with parameters 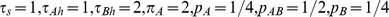. In these plots, the energetic value 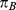 of resource B varies in the interval from 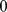 to 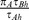 (i.e. 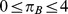). The critical values of 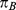 are 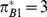; 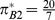; 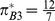; 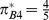. The arrows in each panel indicate the direction of increasing energy intake per unit time at points in the unit square. In each case shown, these arrows lead to a single vertex indicated by the filled in circle which corresponds to the optimal foraging behavior (and unique NE). (a) For 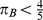, 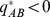 and 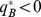. Thus 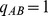 and 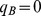. (b) For 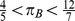, 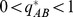 and 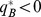. Thus 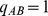 and 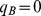. The dashed line denotes 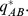 As 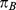 increases, 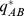 moves to the right until it coincides with the vertical line 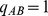 when 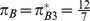. At this critical value of 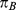 (not shown), all points 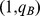 on this vertical line are optimal foraging strategies (and NE). (c) For 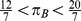, 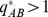 and 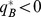. Thus 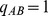 and 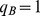. (d) For 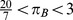, 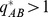 and 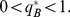 Thus 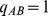 and 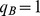. The dashed line denotes 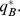 (e) For 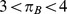, 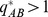 and 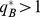. Thus 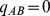 and 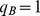.

By Theorem 3 in section Zero-one rule and the Nash equilibrium of Appendix S1, NE correspond to optimal foraging behavior. Thus, the optimal foraging behavior depends critically on the values of 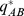 and 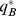. In particular, it is important to know whether these values are between 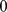 and 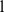, less than 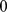 or greater than 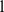. For instance, suppose that 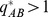 and 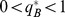. Then, from (10), 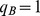 (since 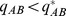) and so 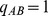 by (8). In this case, the only optimal foraging behavior is to consume 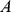 whenever it is encountered and to consume 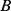 only when it is not encountered simultaneously with 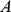. In general, we observe that (i) if 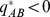, then 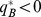, and (ii) if 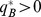, then 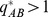. These inequalities constrain the number of possible optimal strategies to 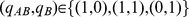. These are the possible optimal strategies among the vertices of the unit square in Figure 4. As we will see, at certain threshold parameter values, two of these vertices can both correspond to optimal behavior. In this case, all points on the edge between these vertices correspond to optimal behavior as well. In particular, the case where 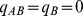 can never occur because the two necessary conditions 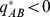 and 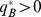 are excluded by (i) and (ii). This intuitive result predicts that if the less profitable resource type 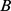 is consumed when encountering both types (i.e., if 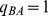 which implies that 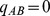), it will always be consumed when encountered alone. Moreover, there is no interior optimal strategy (i.e. there is no NE whereby the consumer exhibits partial diet choice when encountering both resource types simultaneously as well as partial diet choice when encountering resource B on its own) since this requires that both 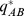 and 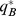 be strictly between 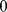 and 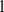, which does not happen for any parameter choice.

It is interesting to analyze dependence of the optimal strategy 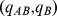 on the energetic value (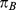) of the less profitability prey. We consider only those energetic values for which B is the less profitable prey type (i.e., 
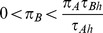
). To this end, we need to know the critical values of 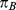 when either 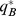 or 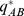 are equal to 0 or 1. Let
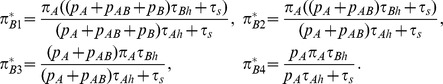



**Figure 5:**
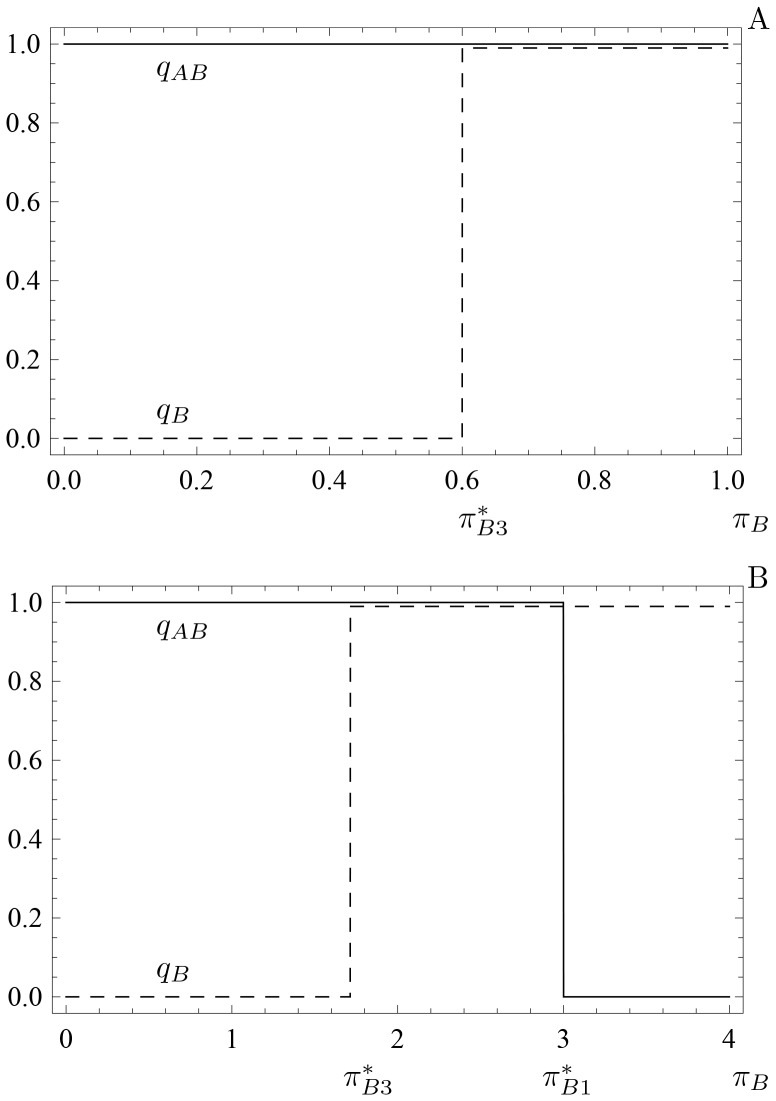
Dependence of the optimal foraging strategy 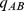 ((8), solid line) and 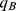 ((10), dashed line) on the energy content of the less profitable prey type B. Panel A assumes a larger handling time of prey type A (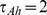, 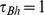), while panel B assumes the opposite case (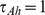, 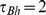). Other parameters 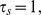 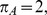 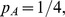 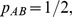 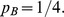

Then 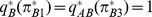 and 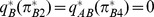. We divide the analysis into two cases. For the first, assume that the handling time of resource A is longer than or equal to that of resource B (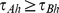). As we assume that resource A is more profitable than B, it follows that the energy content in food items must be larger in resource A (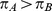) and so 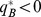 by (9). Thus, the optimal foraging strategy is to consume the 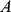 resource when both are encountered (i.e. 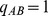). Furthermore, 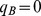 if 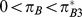 and 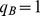 if 
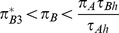
 (i.e. the 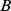 resource is consumed when encountered on its own only if its energy value is sufficiently high). The dependence of the optimal strategy as a function of prey B energetic value is shown in Figure 5A.

The more interesting case where prey A handling time is shorter than prey B handling time (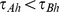; Figure 5B) is analyzed in section Foraging with simultaneous resource encounters of Appendix S1. When energy content of prey B is smaller than 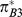 the optimal strategy is 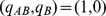. For intermediate energy content satisfying 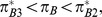 the optimal strategy is 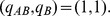 For relatively large energy content 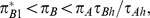 the optimal strategy is 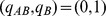.

These results are also included in Figure 4 that in addition provides the direction of increasing energy intake per unit time at all points in the unit square. In all cases analyzed in the previous two paragraphs, the outcome satisfies the zero-one rule (i.e. either always consume a given resource type in a given patch or never consume it) as suggested by [31].

It is particularly interesting to see what happens at the critical values 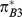 where 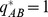, and 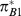 where 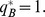 These values correspond to transitions (b)-(c) and (d)-(e) respectively in Figure 4 because the dashed vertical (panel (b)) and horizontal (panel (d)) lines respectively are then on the boundary of the unit square. Straightforward calculations show that
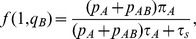
when 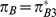. Thus 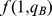 is independent of 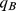 and the optimal foraging behavior is any strategy pair of the form 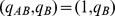 for 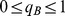.

Similarly, when 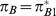,
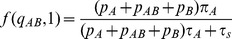
and the optimal foraging behavior is any strategy pair of the form 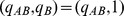 for 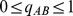. Once again, the zero-one rule must be modified at these critical values. For instance, when 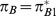, resource B is always consumed under optimal foraging when encountered on its own. However, if both resources are encountered simultaneously, optimal foraging occurs for any preference for the less profitable prey type. In section Zero-one rule and the Nash equilibrium of Appendix S1, the modified zero-one rule states that there is at least one optimal foraging behavior where the corresponding NE is a pure strategy, i.e., where the predator preference for a prey is either 0 or 1. After such modification the zero-one rule holds even at 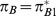 because the (pure) strategy 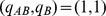 is optimal. This extension of the zero-one rule applies to situations where optimal preferences for prey types as a function of a parameter switch suddenly at some critical values from 1 to 0 or vice versa.

These results can be partially explained through the patch choice model of [31]. Specifically, since patches A and AB have the same maximum profitabilities 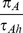 (which is higher than in patch B), both are included in the consumer's diet. However, as shown by [31], this does not mean that the most profitable resource is chosen in patch AB. From (9), we see whether 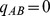 or 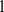 depends both on the ranking of 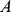 and 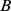 profitabilities (the denominator in (9)) as well as on the difference in energy gain 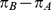 per unit consumed. When resource type A is both more profitable and also has a higher energetic value (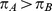), or search time is short, then 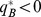 and, consequently, 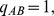 i.e, only resource A will be consumed in patches containing both resource types. Only when type B is energetically more valuable than type A and either search time is long enough, or the probability of encountering patch A and patch AB is low enough, can resource type B be selected when both resources are encountered simultaneously. In this case, resource B will also be consumed when encountered on its own.

### Prey recognition effects

**Figure 6:**
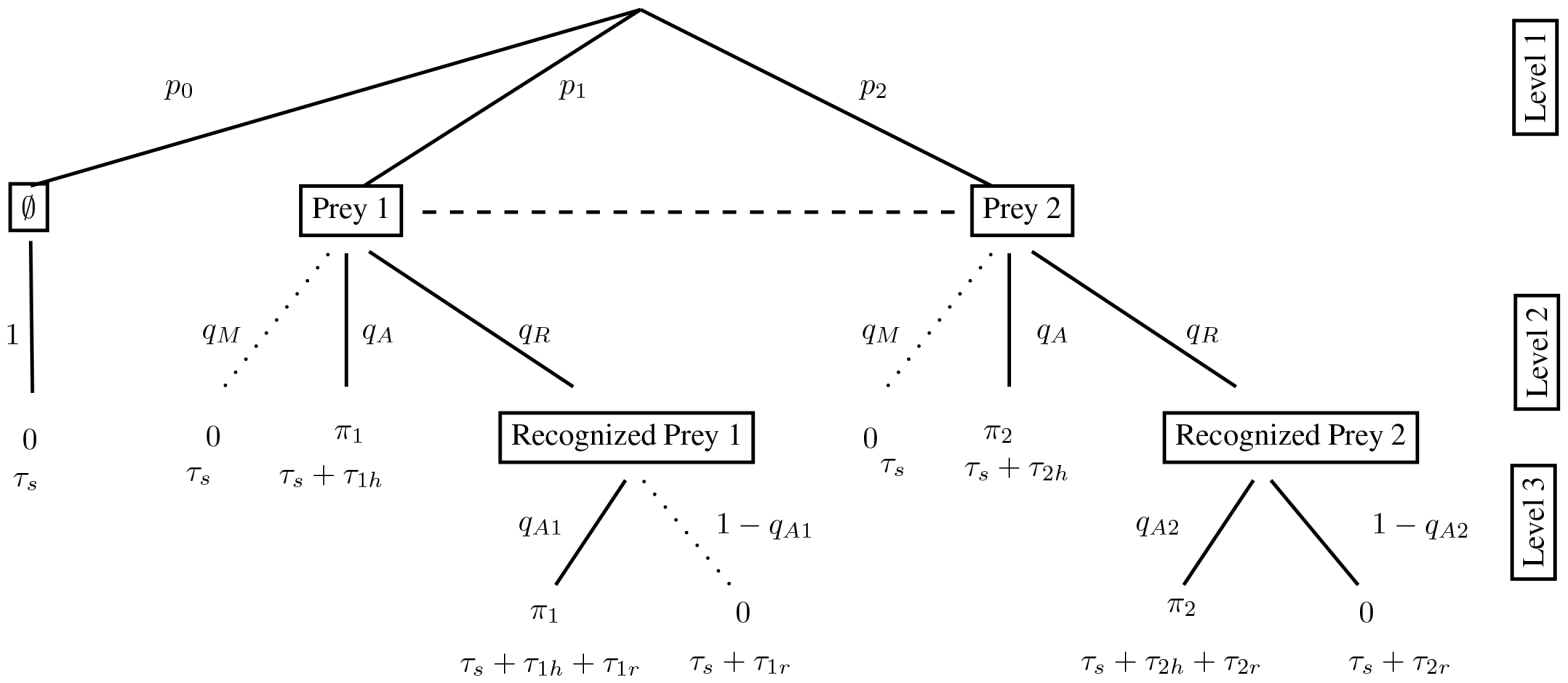
Decision tree for prey recognition game. In the reduced tree, the dotted edges are deleted.

The functional response developed in section Decision trees and functional response for two prey types assumes the predator immediately recognizes the type of prey found during its search and then decides whether or not to attack it. In this section, we model the situation where the predator cannot distinguish the type of prey it encounters unless it is willing to spend extra “recognition” time 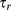 beyond the time required to search the microhabitat. That is, the predator has an option of paying this extra cost to gain information on the prey type encountered before it decides whether to attack. This information is said to be gathered in the facultative sense [32]. Kotler and Mitchell [32] point out instances of facultative information that occur in host-parasite and in mate selection models. They also discuss optimal foraging when information is gathered in the obligate sense (i.e. the predator gathers this information on prey type in the process of handling the prey and has the option of rejecting it at that point). Although we do not consider the model for obligate information in this paper, its decision tree (and analysis of optimal foraging) is simpler than Figure 6 for facultative information.

As in section Decision trees and functional response for two prey types, we assume that the two prey types are distributed among 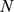 micohabitats with at most one prey in each. To ease notational difficulties, we now label these prey types as species 1 and 2 with densities 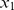 and 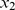 respectively and nutritional values 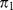 and 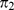 respectively to the predator. If the predator chooses a microhabitat at random, the encounter event distribution (see Figure 6) is the same as in Figure 1 (with our change of notation). In particular, 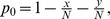 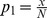 and 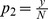.

On finding a prey in a microhabitat, the predator decides immediately whether to attack, move to another microhabitat to begin a new search, or spend recognition time to determine the type of prey encountered. Suppose these choices are taken with probabilities 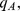 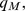 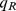 respectively (where 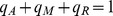). The horizontal dashed line in Figure 6 joining these two encounter events indicates that this decision must be made without knowing the type of prey. Thus, in the terminology of extensive form games [19], the set of these two nodes forms an “information set” of the predator and is represented by a single player in the three-player game corresponding to Figure 6. We remark that the two nodes that form this single information set require only one player because, at both nodes, the information available is the same (the information is that the searching predator encountered a prey).

If the predator decides to spend recognition time to determine the encountered prey is of type 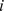, then it must subsequently decide whether to attack this prey or not with probabilities 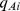 and 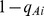 respectively (see the third level of Figure 6). It is not necessary that 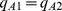. If we assume that the predator is always successful when attacking a prey, the tree diagram is given in Figure 6 where 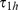 and 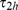 are the handling times for prey of type 1 and 2 respectively. We also assume that the time needed to recognize either type of prey is the same (i.e. 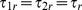). Proceeding as in section Decision trees and functional response for two prey types, the functional response to prey type 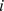 is given by
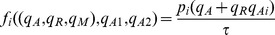
where 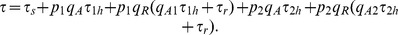 Thus the total predator nutritional value per unit time is
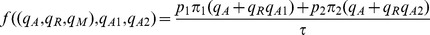
for fixed prey distribution 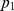 and 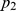.

The optimal predator foraging behavior corresponds to the maximum of 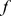 as a function of 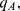 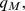 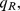 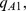 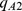. This maximum is considerably harder to determine than in section Decision trees and functional response for two prey types. However, game-theoretic methods to solve for NE are effective at simplifying the analysis. Figure 6 is a three-player foraging game with player 1 representing the predator decision at the two-node information set at level 2 and players 2 and 3 assigned to the respective decision nodes at level 3. From section The Nash equilibria of the prey recognition game of Appendix S1, any strategy 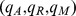 of player 1 with 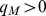 is strictly dominated and so any NE behavior of this player must satisfy 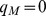. Thus, at the optimal strategy, the predator should never move to another microhabitat when it first finds a prey since, by abandoning this prey, the predator wastes the time spent searching for it. In contrast to section Decision trees and functional response for two prey types where the predator could reject the prey type with low profitability on first encounter, this is not possible here without rejecting the better prey type as well (because upon an initial encounter the predator does not know the prey type).

Since player 1 has strategy of the form 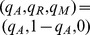 for some 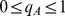, we will denote the NE behavior of player 1 by 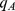 and assume that 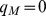 from now on. Section The Nash equilibria of the prey recognition game of Appendix S1 also shows that, if prey type 1 is more profitable than type 2 (i.e. if 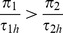 as in section Decision trees and functional response for two prey types, then the predator must attack any prey 1 that it recognizes. We will assume this throughout this section. Thus, we will also assume that 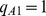 in the decision tree of Figure 6 and analyze the truncated foraging game that eliminates the three edges indicated by dotted lines there.

The reduced tree corresponds to a two-player game with strategy set 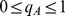 for player 1 at level 2 and 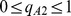 for player 2 representing the predator decision whether to attack a recognized prey 2 at level 3. The energy intake rate is then
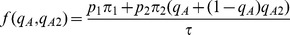
(12)where 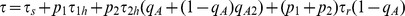.

The NE of this truncated game is easy to determine when 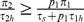. In this situation, the profitability of prey type 2 is at least as high as the nutritional value of only attacking prey type 1 when there is no recognition time (i.e. 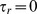). In section Decision trees and the functional response for two prey types (cf. equation (1)), the NE behavior of player 1 is then to attack any prey encountered and this continues to be the NE strategy when recognition time is non-zero. Thus 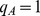 at any NE and, in fact, all NE are of the form 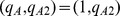 for some 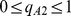 (see section The Nash equilibria of the prey recognition game of Appendix S1 for the formal derivation). This corresponds to the predator being opportunistic (sensu [32]). Note that, if the predator immediately attacks an observed prey, the decision whether to attack after recognizing the type of prey is no longer relevant since this choice is never needed.

For the remainder of this section, assume that the profitability of resource 2 is lower than is the mean energy intake rate obtained when feeding on the more profitable prey type only (i.e., 
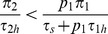
). In this case, the predator should consider whether to determine the prey type it encountered, because including the less profitable prey type in its diet may decrease the mean energy intake rate.

To calculate the NE behavior, we proceed as in section Foraging with simultaneous resource encounters. From section The Nash equilibria of the prey recognition game of Appendix S1, the best response of player 1 to a given strategy 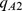 of player 2 is
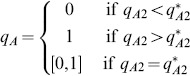
(13)where
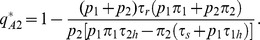



Conversely, the best response of player 2 to a given strategy 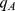 of player 1 is
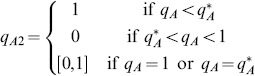
(14)where
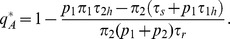



That is 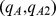 is a NE foraging behavior if and only if it satisfies (13) and (14). We remark that 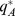 and 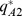 are both less than 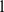, and 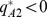 when 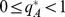.

**Figure 7:**
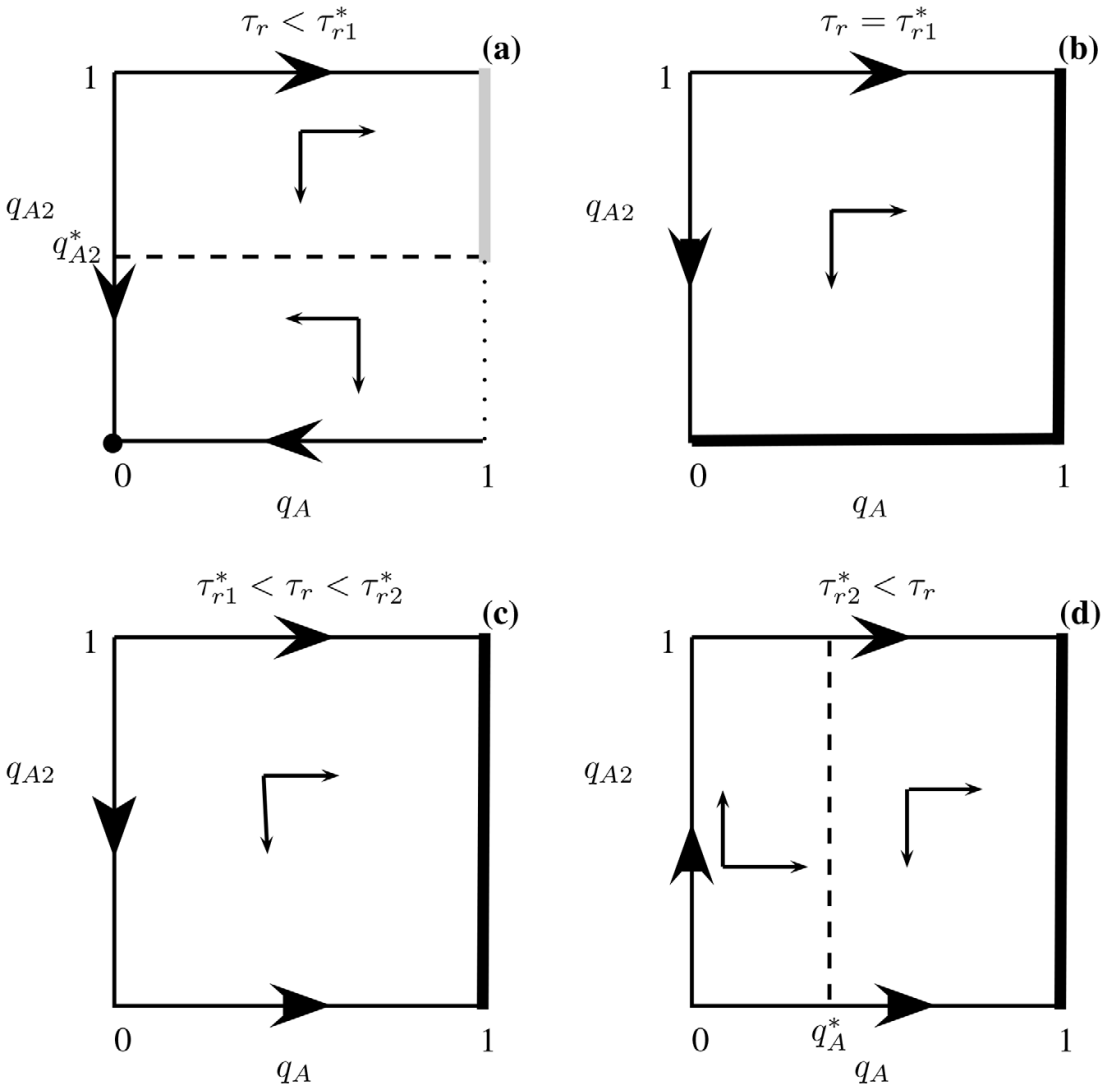
Qualitative outcomes of the optimal foraging strategy (13) and (14) for increasing recognition time 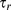. Panel (a) assumes 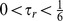 for which 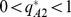 and 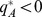. The optimal foraging strategy is at 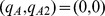 (i.e. always pay the cost of recognition and then never attack the less profitable prey type) and the NE component (shown as the gray line segment) 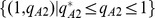 (corresponding to the NE outcome of attacking immediately) is suboptimal. In each of the other three panels, the (union of the) thick edges forms a strict equilibrium set (SES, for definition see section Zero-one rule and the Nash equilibrium of Appendix S1) that is the globally stable evolutionary outcome. Panel (b) assumes 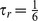, 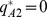 and 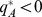. The union of the two edges 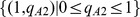 and 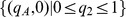 forms one NE component corresponding to optimal foraging behavior. Panel (c) assumes 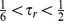, 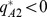 and 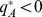. The edge 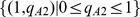 forms a NE component corresponding to optimal foraging behavior. Panel (d) assumes 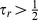 for which 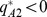 and 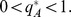 The edge 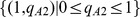 forms a NE component corresponding to optimal foraging behavior. The arrows in each panel indicate the direction of increasing energy intake per unit time at points in the unit square. Other parameters 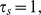 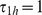, 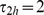, 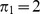, 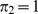 and 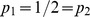.

When recognition time, 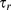, is small (
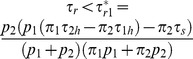
), 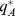 is negative and 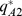 is positive (Figure 7(a)). There are then two possible NE outcomes; namely, attack any encountered prey immediately corresponding to the NE 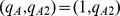 where 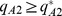 (shown as the gray segment of the line in Figure 7(a)) or never attack immediately and then only attack prey type 1 when recognized (with NE 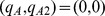, the solid dot in Figure 7(a)). From section Zero-one rule and the Nash equilibrium of Appendix S1, the optimal foraging behavior must be a NE outcome but because our decision tree has three levels, every NE may not be an optimal strategy. However, even in this case finding all NE substantially simplifies the problem of finding the optimal strategy, because it is now enough to evaluate function 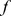 given by (12) only at these NE points. Moreover, if there is a NE component (such as the gray segment in Figure 7(a)) the value of 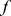 at any point in this segment must be the same. By evaluating 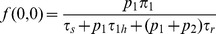 and 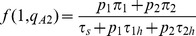, we find 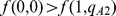 and so the optimal behavior is to never attack immediately and then only attack prey type 1 when recognized. As recognition time increases, 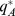 increases and 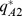 decreases. However, the NE outcomes and optimal behavior remain the same as long as 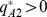. Optimal predator behavior is then either described as being selective [32] or as being intentional [33].

For recognition time satisfying 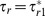, 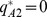 while 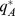 remains negative and so all strategy pairs of the form 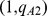 and 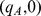 are NE (Figure 7(b)). Moreover, each corresponds to an optimal foraging strategy since 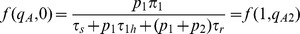 in this case.

For still larger recognition times, 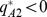, thus 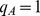 at any NE. When 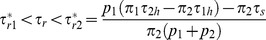, 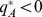 and the corresponding optimal foraging behavior is shown in Figure 7(c), while for recognition time larger than 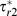, 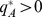 (Figure 7(d)). In both cases, the NE strategy pairs are of the form 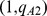 and these all yield optimal foraging behavior.

### Game theory and evolutionary outcomes for the prey recognition game

The existence of suboptimal NE in the prey recognition game makes the interesting question considered briefly in section Decision trees and extensive form games even more important here; namely, how does the predator manage to learn its optimal behavior and avoid suboptimal equilibrium behavior. This type of question (on the so-called equilibrium selection problem [30]) is commonly studied in evolutionary game theory where individual behaviors evolve in such a way that strategies with higher payoff become used more frequently. There are several standard models that examine the evolutionary outcome of these behaviors changing over time [29], [34].

The evolutionary outcome is clear for all choices of parameters in the two diet choice models of sections Decision trees and the functional response for two prey types and Foraging with simultaneous resource encounters (see arrows in Figures 2 and 5, respectively). These arrows indicate the direction of increasing energy intake rate (e.g. in Figure 4, this rate increases as 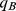 is used more frequently if and only if the vertical arrow is pointing upward). In all cases, the predator learns to use the NE strategy that corresponds to the optimal behavior for the foraging games of Figures 1 and 3 respectively. (This is true for Figure 2b as well since the arrows lead to some point on the vertical side of the unit square with 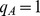, all of which correspond to optimal behavior in this threshold case when 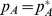.)

The evolutionary outcome is also clear for the prey recognition game of this section when recognition time is large from Figure 7. Specifically, for 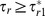 (panels (b), (c) and (d)), the predator will evolve to a strategy on an edge consisting of NE points which correspond to optimal foraging. In the language of evolutionary game theory, this set of NE forms a globally stable set that attracts any initial predator behavioral choice as long as behaviors evolve in the direction of increasing energy intake rate.

However, for short prey recognition time (i.e. 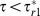 with 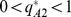 in Figure 7(a)), the NE 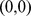 (corresponding to the optimal foraging behavior of always spending the time to recognize the type of prey encountered and then only attacking prey of type 1) may not be globally stable. If the predator initially attacks recognized prey 2 with probability greater than 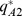, behavior may evolve to a point in the suboptimal NE component where 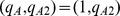 with 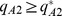 (i.e. to a point on the gray line segment in Figure 7(a)). That is, the predator may become trapped at this suboptimal behavior, especially if evolution increases the strategy of attacking immediately faster than it decreases the strategy of attacking recognized prey 2 (i.e. if the arrow to the right in the top half of Figure 7(a) is much bigger than the one pointing down).

The situation depicted in Figure 7(a) is remarkably similar to that of the two-player extensive form Chain store game [19], [35] (also known as the Ultimatum mini-game [36] or the Entry deterrence game [37]). In the large literature on this game, it is often argued that the evolutionary outcome will be the point 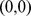) since neutral drift near the suboptimal NE component will inevitably lead at some time to the strategy choice shifting to 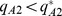 after which selection will quickly lead to 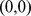. To see this, consider a point on this gray line segment. If the predator decides once in a while to spend some time to recognize the type of prey it encounters, its strategy will move to the left of the segment. As strategies with higher payoff are then to the right and down in the vicinity of the line segment, it is likely that 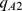 will regularly decrease until it reaches the lower end of the segment. Any further strategy experimentation on the part of the predator will lead to 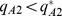, after which the only evolutionary outcome can be 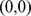. In terms of evolutionary game theory again, the suboptimal NE component is not stable whereas the optimal NE is.

In summary, the optimal foraging behavior is selected in the prey recognition game as the NE component that is the stable outcome of the evolutionary learning process whether or not prey recognition time is short (i.e. for arbitrary 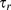). The analysis of optimal foraging theory for this example illustrates anew the potential of game-theoretic methods to gain a better understanding of issues that arise in behavioral ecology.

## Discussion

In this article, we develop a game-theoretic approach for constructing functional responses in multi-prey environments and for finding optimal foraging strategies based on these functional responses [9], [20]. The approach here is based on methods from extensive form games [18], [19]. The importance of these game-theoretic approaches for functional response is two-fold. First, decision trees similar to those used in extensive form games are a natural way to describe details of predator behavior based on the sequence of choices the predator makes at different decision points. This facilitates writing down the corresponding functional response. Second, we show that optimal foraging behavior that maximizes energy intake per unit time can be determined by solving the underlying foraging game for its Nash equilibrium. We documented these game theory methods through three examples: the classical diet choice model, simultaneous encounter with prey, and a model in which recognition time is considered. We remark that, although the calculation of the optimal foraging behavior in the first example is straightforward, it is not as easy in the last two cases where our game theory methods lead readily to the solution.

Decision trees are often used in evolutionary ecology to describe possible decision sequences of individuals in biological systems [28], including models of kleptoparasitism [38] and of producers and scroungers [39]. They have been used less often in connection with functional responses, even though the predation process can be conveniently described by such trees (e.g., [26], [40], [41]). Optimal foraging behavior that maximizes animal fitness is then often described as a sequence of single choices at each decision node faced by the predator. Such outcomes are reminiscent of those found by applying the backward induction technique to extensive form games that also chooses one strategy at each decision node [18], [19]. However, there are essential differences. Specifically, under backward induction, the optimal choice at such a node depends only on the comparisons of payoffs along paths following this node. Unfortunately, the time constraint in our foraging game means that decisions at one node have payoff consequences as to what is optimal at another node, a connection between these decision nodes of the tree that has no counterpart in extensive form games. That is, the payoff concept for “foraging games” such as Figure 1 combines both the nutritional values and the duration of each activity given at all the end nodes of the decision tree. On the other hand, as shown in all three examples, the extensive form technique connected to backward induction of forming the reduced decision tree by truncating those paths corresponding to dominated strategies remains an effective means of considerably simplifying the NE analysis.

Dynamic programming (a form of backward induction) has also been used to find optimal foraging behavior [23], [42]. Specifically, the approach developed by Houston and McNamara [23] shows that the optimal foraging strategy must maximize the difference between the expected energy intake during a single renewal cycle and the product of the mean optimal energy intake rate and the duration of the cycle. This approach specifies the optimal choice at each decision node provided the energy intake rate under the optimal strategy is known. Since the optimal choice in one part of the decision tree then requires knowing the overall optimal strategy, the solution is typically obtained by numerical iteration.

Instead, the approach we take in this article avoids such numerical methods by solving the game analytically. In this game, virtual players (also called agents) are associated with each decision point. These players are virtual because their payoff is derived from the functional response of a single individual only. Nevertheless, these players play a game because their decisions are linked, one player's optimal strategy depends on the other players' decision. We showed that solving this game by finding all the Nash equilibria will lead to the optimal foraging strategy. In those cases where some NE are not optimal foraging strategies, we showed it is easy to select the optimal ones among them by calculating their mean energy intake rate. Even when the game has infinitely many Nash equilibria that form a segment of a line (such Nash equilibrium components often arise in extensive form games), we showed that the energy intake rate at all these Nash equilibria will be the same. This means that once there are a finite number of isolated Nash equilibria points or Nash equilibrium components, finding the optimal strategy corresponds to comparing a finite number of values, which is trivial.

We documented these game-theoretic methods by applying them to three examples. The classic diet choice model with two prey types where predators encounter prey sequentially was considered first since it has been historically analyzed without game theory and yet provides an informative introduction to our new approach. Then we moved to a more complicated situation where a searching predator can simultaneously encounter both prey types [31], [43]. These authors showed that under simultaneous encounter the predictions based on the prey profitabilities (i.e., energy content over handling time) are not sufficient to predict the optimal foraging strategy. In fact, the optimal foraging strategies can be quite complicated as they depend now also on the relation between the energy content in different food types. In particular, Figure 5A shows that when the less profitable prey type 2 contains also less energy than the more profitable prey type 1, then the more profitable prey type 1 will be selected when both prey types are encountered. However, when the energy content of the less profitable prey type is large (but still small enough that prey type 2 continues to be less profitable), it will be preferred when both prey types are encountered (Figure 5B). The solid line in Figure 5B shows the preference for prey type A when encountered with prey type B. When this preference switches from 1 to 0 above 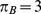, predator preference for prey type B when encountered with prey type A switches from 0 to 1. All possible optimal foraging strategies as a function of the alternative prey type energy content are shown in Figure 4. In particular, it cannot happen that the less profitable prey type is included in the predator's diet when encountered simultaneously with the more profitable prey type but not taken when encountered alone.

The last model discussed in this article examines whether a predator should spend time to recognize which type of prey it encountered before deciding whether to attack the prey or not [32]. This example is more complex for several reasons, including the fact that the corresponding decision tree now has three different levels (whereas the previous two examples are described by two-level trees). While the NE corresponds exactly to the optimal strategy in two-level decision trees, this is not the case here. When recognition time is small, we show that there are NE of the optimal foraging game that lead to suboptimal foraging (Figure 7(a)). However, these suboptimal NE are easy to exclude by equilibrium selection techniques borrowed from evolutionary game theory [30]. Specifically, optimal foraging is always given by the unique NE outcome that corresponds to the stable equilibrium point (or set of equilibrium points) as the predator learns its optimal strategy (i.e. as its strategy evolves in the direction of the arrows in Figures 2, 4, 7).

This method taken from evolutionary game theory to determine optimal foraging behavior differs from the more traditional approach based on the (modified) zero-one rule. This latter approach can be applied to the prey recognition game. Kotler and Mitchell [32] show that the zero-one rule yields just two possible optimal outcomes: either complete opportunism or completely selective. Instead of analyzing for the effects of increasing recognition time as we have done, they concentrate on what happens when the abundance of the less profitable prey increases (which, in our notation, means 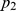 increases). They emphasize the somewhat counterintuitive result that, with low abundance, the less profitable prey is excluded from the diet. At intermediate abundances it is included, and then with high abundance it is excluded again.

Game-theoretic methods play an important role in the traditional approach as well. Specifically, because the energy intake rate is the same at all points of the NE component, we need to compare only two numbers; the energy intake rate at any point of the NE component (the gray line segment in Figure 7(a)) and the energy intake rate at the other NE point 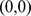. Our analysis shows that, when recognition time is small, the optimal foraging strategy is to always pay the extra time to recognize the encountered prey type (i.e., never attack the encountered prey item immediately 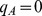, Figure 7(a)) and to include it in the diet if it is the more profitable prey type (i.e., not to include the alternative prey type 2, 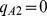). As the recognition time increases, the optimal foraging strategy is not to waste time recognizing the encountered prey type (Figure 7c, d). In this case, all encountered prey types are included in predator's diet and so 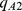 is not uniquely defined. That is, since all encountered prey are immediately included in predator's diet, the question whether to include the recognized prey type in the diet becomes irrelevant and so the preference for the alternative prey type is any number between 0 and 1.

For the three optimal foraging games modeled in this paper, the predator's encounter probabilities with different prey types do not change over the system's renewal cycle. In particular, there are no interactions among predators, such as competition for the same prey, that may alter the length of this cycle as the predator's behavior in these interactions changes. On the other hand, interactions among predators can be added to their decision trees. Our analysis of optimal foraging behavior through extensive form game-theoretic methods can then be generalized to the resultant multi-level trees, an important area of future research.

## Supporting Information

Appendix S1 The first section of the Appendix, Decision trees and the functional responses, describes a general approach to construct functional responses from decision trees. The second section, Zero-one rule and the Nash equilibrium, generalizes the classical zero-one rule of the optimal foraging theory derived for the multi-prey Holling type II functional response to a more general functional responses. This section also shows how the zero-one rule relates to the Nash equilibrium of the underlying optimal foraging game. Appendix Foraging with simultaneous resource encounters derives the Nash equilibrium strategy (8), (10) and Appendix The Nash equilibria of the prey recognition game derives the Nash equilibrium (13), (14).
(PDF)
