# Supplementary material for: Game-Theoretic Methods for Functional Response and Optimal Foraging Behavior
Source: PLoS One. 2014 Feb 28;9(2):e88773. doi: 10.1371/journal.pone.0088773 (PMC3938838; doi:10.1371/journal.pone.0088773)
Supplement: Appendix S1 — The first section of the Appendix, Decision trees and the functional responses, describes a general approach to construct functional responses from decision trees. The second section, Zero-one rule and the Nash equilibrium, generalizes the classical zero-one rule of the optimal foraging theory derived for the multi-prey Holling type II functional response to a more general functional responses. This section also shows how the zero-one rule relates to the Nash equilibrium of the underlying optimal foraging game. Appendix Foraging with simultaneous resource encounters derives the Nash equilibrium strategy (8), (10) and Appendix The Nash equilibria of the prey recognition game derives the Nash equilibrium (13), (14). (PDF) [file pone.0088773.s001.pdf]

Game-theoretic methods for functional response and optimal foraging behavior

Ross Cressman[1], Vlastimil Křivan[2,*], Joel Brown[3] József Garay[4]

Appendix S1: Appendix

Decision trees and the functional responses

Decision trees for functional responses describe predator actions at each decision point. When applied to functional responses, these trees must have at least two stages. The first stage describes the distribution of encounter events between predator and prey, (cf. Level 1 in Figure 1). In general, let us denote the different encounter events by E_i and the probability that E_i occurs by $P(E_i)$. Since we also include events when the predator does not encounter any prey, $\sum_i P(E_i) = 1$. Furthermore, let α_k give the predator's possible actions (i.e. what the predator does) in encounter E_i and $s(\alpha_k \mid E_i)$ be the predator's conditional strategy of using action α_k when in encounter E_i. We stress here that actions α_k are specific for each encounter event E_i and so are more formally denoted as α_{ki}. To simplify notation, the second index is omitted throughout the article. Then, for each i, $P[s(\alpha_k \mid E_i)] \geqq 0$ and $\sum_k P[s(\alpha_k \mid E_i)] = 1$ where $P[s(\alpha_k \mid E_i)]$ is the probability of using conditional strategy $s(\alpha_k \mid E_i)$ in event E_i. Finally, the predator's success at killing its prey may also depend on the encounter event E_i and on its action α_k. Let $\kappa(\alpha_k \mid E_i)$ denote the probability the predator is successful, which is 0 if α_k is an action that does not attack a prey and is a number between 0 and 1 otherwise.

For the general modeling approach, we now introduce the "activity distribution" of the predator. An activity event A_ℓ is given by an encounter event E_i, an event specific action α_k and whether or not the predator kills its target (i.e., $A_\ell = \{E_i, \alpha_k, \kappa(\alpha_k \mid E_i)\}$). Taken together, all such events form a partition of the *total event set.* That is, every activity event is included in the union of the A_ℓ and two different A_ℓ and $A_{\ell'}$ are mutually exclusive. The activity distribution answers the three questions posed at each stage of the predation process: encounter, predator's decision, predator's success.

The probability P_ℓ of activity event A_ℓ is given through the information above. For example, if A_ℓ is the encounter event E_1 combined with action α_1 and the predator kills the prey, then $P_\ell = P(E_1)\, P[s(\alpha_1 \mid E_1)]\kappa(\alpha_1 \mid E_1)$. Since the functional response is based on the number of prey killed by the predator per unit time, we must also consider the duration τ_ℓ of each activity event A_ℓ. The activity distribution

together with the duration of these events define one renewal cycle. The result in the following paragraph from renewal theory (e.g., [1–3]) is essential to calculate the multi-prey functional response in our model.

Suppose that the activity distribution is constant over a given time interval T. Then the average number of A_{ℓ_0} activity events per unit time, φ_{ℓ_0}, is given by

$$\varphi_{\ell_0} := \frac{P_{\ell_0}}{\sum \tau_\ell P_\ell}. \tag{S1}$$

Suppose that the predator chooses a habitat at random and let I be the set of all activity events when the predator kills a particular prey type. Then, the expected number of this type of prey killed per unit time (i.e. the functional response to this type of prey) is $\sum_{\ell \in I} \varphi_\ell$.

Predator energy intake rate functions are often generated in optimal foraging theory [2, 3] using renewal theory. The optimal predator strategy then maximizes the expected energy gain per renewal cycle divided by the expected time of the renewal cycle. The approach taken above is closer to that used by Charnov and Orians [4] who also consider the distribution of predator-prey encounter events and introduce tree diagrams to describe the possible predator activities for each such event. This approach provides a straightforward procedure to obtain predator functional responses as illustrated by examples in this article. The renewal theorem used here assumes that the time horizon is infinite. When it is finite, the precise statement from probability theory is that the expected number of A_{ℓ_0} activity events observed in a time interval of length T is somewhere between $\frac{TP_{\ell_0}}{\sum \tau_\ell P_\ell}$ and $\frac{(T+\tau_{\max})P_{\ell_0}}{\sum \tau_\ell P_\ell}$ where $\tau_{\max} := \max\{\tau_\ell\}$, as proven by Garay and Mori [5] using Wald's equation [6]. Thus, the average number of A_{ℓ_0} activity events per unit time is essentially given by $\frac{1}{T} \frac{TP_{\ell_0}}{\sum \tau_\ell P_\ell}$ (i.e. by φ_{ℓ_0} in (S1) when T is sufficiently large relative to $\tau_{\max}$.

For both the simultaneous encounter and the classic (i.e. non-simultaneous) models of the main text, the activity distribution is given at the second level terminal nodes of the two-level decision tree (Figures 1 and 3). The prey recognition game (Figure 6) requires a third level to describe the predator activity distribution since the predator's possible actions in a given encounter event depends on its own second level decision of whether to spend time recognizing the type of prey it encountered before deciding whether to attack. This decision tree also illustrates a situation where the predator must take the same action at different encounter events (specifically at the level 2 information set of Figure 6). Both of these properties of the prey recognition game fit the general framework developed in this Appendix (since the activity distribution is still given by the terminal nodes of the decision tree) and so the functional response can be calculated through (S1). Similarly, the decision trees of Figures 1 and 2 can be easily generalized to

multi-prey models by adding more encounter and activity events at levels 1 and 2 respectively.

Although all three examples in the main text assume that the predator is always successful when it decides to attack a prey in a given encounter event (i.e. $\kappa(\alpha_k \mid E_i) = 1$ if α_k is the action to attack a particular type of prey in encounter event E_i), the decision tree approach remains applicable when this is not the case. For example, if the predator is not always successful when attacking a prey it encounters of a given type (say, prey A) in Figure 1, another level can be included in the tree to account for this by replacing the terminal node after q_A by two branches with probabilities $\kappa(\alpha_1 \mid E_1)$ and $1 - \kappa(\alpha_1 \mid E_1)$. In the terminology of extensive form games, the node following q_A is now a "move by nature" and, since there are no predator choices following this node, the tree can be truncated by deleting these two branches (i.e. by returning to the tree of Figure 1). However, the energy intake and duration time at this truncated node are altered. For instance, if the predator energy intake corresponding to the two deleted branches are π_A and 0 respectively with duration times $\tau_s + \tau_{Ah}$ and τ_s, then the energy intake of attacking prey A becomes $\pi_A \kappa(\alpha_1 \mid E_1)$ and its duration time $(\tau_s + \tau_{Ah})\kappa(\alpha_1 \mid E_1) + \tau_s(1 - \kappa(\alpha_1 \mid E_1)) = \tau_s + \tau_{Ah}\kappa(\alpha_1 \mid E_1)$.

Zero-one rule and the Nash equilibrium

The predator energy intake f per unit time corresponding to the decision tree approach developed in this paper is a rational function of the form

$$f(q_1, q_2, \cdots, q_N) = \frac{P(q_1, q_2, \cdots, q_N)}{Q(q_1, q_2, \cdots, q_N)}$$

when there are N information sets in the tree. Here q_i for fixed $1 \le i \le N$ is an element of the set of mixed strategies Δ^i (i.e. q_i is a probability vector whose components are nonnegative and sum to 1) that correspond to the possible choices at information set i. For example, $\Delta^1 \equiv \{(x_1, x_2, \cdots, x_m) \mid \sum_{j=1}^{m} x_j = 1, x_j \ge 0\}$ is the $m-1$ dimensional strategy simplex when information set 1 has m possible choices. P and Q are polynomials that are linear in the components of each q_i and Q is positive for all $(q_1, q_2, \cdots, q_N) \in \Delta \equiv \Delta^1 \times \Delta^2 \times \cdots \times \Delta^N$. To ease notational complexities, we will assume that $N = 3$ in all proofs in this Appendix and write f as $f(x, y, z)$ where $x \in \Delta^1$, $y \in \Delta^2$ and $z \in \Delta^3$. Although the proofs of the results in this Appendix are given for $N = 3$, they can all be extended to show the results remain true for arbitrary N.

For all of the decision trees analyzed in the main text of this paper, $N \le 3$. In fact, each reduced tree in the main text has $N \le 2$ with Δ^1 and Δ^2 one-dimensional (e.g. $\Delta^1 \equiv \{(x, 1-x) \mid 0 \le x \le 1\}$). In the

decision trees of Figure 1 and Figure 3, the consumer has complete information at each of its decisions nodes (i.e. the information sets are singleton sets and so the number of information sets coincides with the number of decision nodes). For example, in the reduced tree of Figure 3, there are two decision nodes denoted as B and AB. Individuals at each of these two nodes have complete information about the resources encountered and can make one of two choices based on this knowledge. The situation is different in the prey recognition game shown in Figure 6. Here, upon encountering a prey, the predator does not know the prey type. So, the nodes denoted as Prey 1 and Prey 2 combine to form one information set. The other decision node denoted Recognized Prey 2 in the reduced tree is a singleton information set with two choices (i.e. whether or not to attack prey type 2).

Theorem 1 *Optimal foraging behavior occurs at one of the vertices of* $\Delta^1 \times \cdots \times \Delta^N$.

Proof. Assume that $N = 3$. We need to show that, for some choice of vertices $x^* \in \Delta^1$, $y^* \in \Delta^2$ and $z^* \in \Delta^3$, $f(x^*, y^*, z^*) \geq f(x, y, z)$ for all $(x, y, z) \in \Delta^1 \times \Delta^2 \times \Delta^3$.

Since f is a continuous function on a compact set, it attains its maximum value at some point $(x^*, y^*, z^*) \in \Delta^1 \times \Delta^2 \times \Delta^3$. Suppose that x^* is not a vertex of Δ^1. Then $x^* = (x_1^*, \cdots, x_m^*)$ where $0 < x_j^* < 1$ for at least two different values of j (say $j = 1$ and 2). Consider $f((x_1^*+\varepsilon, x_2^*-\varepsilon, \cdots, x_m^*), y^*, z^*)$ for $-x_1^* \leq \varepsilon \leq x_2^*$. Since $(x_1^* + \varepsilon, x_2^* - \varepsilon, \cdots, x_m^*) \in \Delta^1$, $f((x_1^* + \varepsilon, x_2^* - \varepsilon, \cdots, x_m^*), y^*, z^*) \leq f(x^*, y^*, z^*)$ and so $\frac{\partial f((x_1^*+\varepsilon, x_2^*-\varepsilon, \cdots, x_m^*), y^*, z^*)}{\partial \varepsilon} |_{\varepsilon=0}= 0$. But $f((x_1^* + \varepsilon, x_2^* - \varepsilon, \cdots, x_m^*), y^*, z^*) = \frac{P((x_1^*+\varepsilon, x_2^*-\varepsilon, \cdots, x_m^*), y^*, z^*)}{Q((x_1^*+\varepsilon, x_2^*-\varepsilon, \cdots, x_m^*), y^*, z^*)}$ where P and Q are polynomials that are linear in ε. That is $f((x_1^* + \varepsilon, x_2^* - \varepsilon, \cdots, x_m^*), y^*, z^*) = \frac{a\varepsilon+b}{c\varepsilon+d}$ with $c\varepsilon + d > 0$ and $\frac{\partial f}{\partial \varepsilon} = \frac{ad-bc}{(c\varepsilon+d)^2} = 0$ for all $-x_1^* \leq \varepsilon \leq x_2^*$. Thus f is a constant function of ε and so both $f((x_1^* + x_2^*, 0, x_3^* \cdots, x_m^*), y^*, z^*)$ and $f((0, x_1^* + x_2^*, \cdots, x_m^*), y^*, z^*)$ equals $f(x^*, y^*, z^*)$ (i.e. f has its maximum at both these points). Thus (x^*, y^*, z^*) can be replaced by either one of these points and the process continued until one component of x^* equals 1 and all the others are 0. That is, x^* is a vertex of Δ^1. A similar process applied to Δ^2 and Δ^3 implies that y^* and z^* can be taken as vertices of Δ^2 and Δ^3 respectively.

■

By Theorem 1, to determine the optimal foraging outcome, we can evaluate f at all the vertices of $\Delta^1 \times \Delta^2 \times \cdots \times \Delta^N$ and take the largest of these values. In our models, the corresponding vertex (or each of the vertices) satisfies the zero-one rule (i.e. either always consume the resource of a given type in all encounters of this type or never consume it) and corresponds to a pure strategy choice for

each agent in the $N-$player agent normal form game that assigns a separate player to each of the N information sets of the decision tree. As we have seen in the main text, optimal foraging behavior can also occur when agents use mixed strategies $(q_1, q_2, \cdots, q_N) \in \Delta^1 \times \Delta^2 \times \cdots \times \Delta^N$. In sections Decision trees and the functional response for two prey types and Foraging with simultaneous resource encounters, only pure strategies can correspond to the optimal behavior for generic parameter values; whereas section Prey recognition effects illustrates a generic game whose optimal behavior may occur at mixed strategies. The following Theorem relates the optimal foraging behavior to the solution of the $N-$player game. The second part of the theorem introduces the concept of a strict equilibrium set (SES). By definition [7], E is a SES if it is a set of NE $(x^*, y^*, z^*) \in \Delta^1 \times \Delta^2 \times \Delta^*$ such that, if $f(x, y^*, z^*) = f(x^*, y^*, z^*)$ (respectively, $f(x^*, y, z^*) = f(x^*, y^*, z^*)$ or $f(x^*, y^*, z) = f(x^*, y^*, z^*)$) for some $(x^*, y^*, z^*) \in \Delta^1 \times \Delta^2 \times \Delta^3$ with $x \in \Delta^1$, $y \in \Delta^2$ or $z \in \Delta^3$, then $(x, y^*, z^*) \in E$ (respectively, $(x^*, y, z^*) \in E$ or $(x^*, y^*, z) \in E$). The concept of SES generalizes the idea of a strict NE. In fact, if a SES is a singleton set (i.e. contains exactly one point), then this point is a strict NE. The general concept is important for optimal foraging, because it includes the situation where, at a critical population density, the optimal strategy is not uniquely defined. For example, in the case of the classic diet choice model this happens when the encounter rate of a searching predator with the more profitable prey type is given by (2).

Theorem 2 *(a) If optimal foraging behavior occurs at $(q_1, \cdots, q_N) \in \Delta^1 \times \cdots \times \Delta^N$, then $(q_1, \cdots, q_N)$ is a Nash equilibrium of the $N-$player agent-normal form of the optimal foraging game.*
(b) The set E of all strategies $(q_1, \cdots, q_N) \in \Delta^1 \times \cdots \times \Delta^N$ corresponding to optimal foraging behavior is a SES of the $N-$player agent-normal form of the optimal foraging game.

Proof. (a) Assume that $N = 3$ and optimal foraging occurs at $(x^*, y^*, z^*) \in \Delta^1 \times \Delta^2 \times \Delta^3$. Since the agent-normal form has payoff $f(x, y, z)$ for each player when the strategy used by players 1, 2 and 3 are x, y and z respectively, (x^*, y^*, z^*) is a NE if and only if $f(x, y^*, z^*) \leq f(x^*, y^*, z^*)$, $f(x^*, y, z^*) \leq f(x^*, y^*, z^*)$ and $f(x^*, y^*, z) \leq f(x^*, y^*, z^*)$ for all $(x, y, z) \in \Delta^1 \times \Delta^2 \times \Delta^3$. These inequalities are obvious from $f(x^*, y^*, z^*) \geq f(x, y, z)$ for all $(x, y, z) \in \Delta^1 \times \Delta^2 \times \Delta^3$.

(b) It is obvious that E satisfies the conditions of a SES. For example, $(x, y^*, z^*) \in E$ when $f(x, y^*, z^*) = f(x^*, y^*, z^*)$ since (x, y^*, z^*) is then an optimal foraging behavior and so in E by its definition.

■

In all three foraging examples considered in the main text, there is a unique SES for every choice of model parameters. In particular, the suboptimal NE component E' in the prey recognition game when recognition time is short is not a SES since all points on the vertical line with $q_A = 1$ in Figure 7(a) are best response to points in E' but not all of them are in E'. Thus, optimal foraging behavior is characterized either by solving for the SES or by finding the asymptotically stable set of NE under the evolutionary dynamics. The equivalence between these two game-theoretic techniques is not surprising here given the fact that they are also equivalent for the standard evolutionary dynamics (i.e. the replicator equation) used in general multi-player extensive form games [7].

In the special case that all predator information sets are at level 2 in the decision tree (e.g. the two optimal foraging game of section Decision trees and the functional response for two prey types and the simultaneaous encounter game of section Foraging with simultaneous resource encounters), the following theorem gives an even closer connection between optimal foraging behavior and Nash equilibrium.

Theorem 3 *Suppose that there are no predator decision points that depend on the outcome of predator choices at previous predator decision points. Then optimal foraging behavior occurs at $(q_1, \cdots, q_N) \in \Delta^1 \times \cdots \times \Delta^N$ if and only if $(q_1, \cdots, q_N)$ is a Nash equilibrium of the $N-$player agent-normal form of the optimal foraging game. Furthermore, a set E is a SES if and only if it is the set of all strategies $(q_1, \cdots, q_N) \in \Delta^1 \times \cdots \times \Delta^N$ corresponding to optimal foraging behavior.*

Proof. These statements are straightforward consequences of the fact

$$f(q_1, q_2, \cdots, q_N) = \frac{P(q_1, q_2, \cdots, q_N)}{Q(q_1, q_2, \cdots, q_N)}$$

where P and Q are linear polynomials in the components of all the q_i (e.g., when $N = 2$, $P(x, y)$ has no term of the form $x_1 y_1$). We remark that this contrasts with the prey recognition game of section Prey recognition effects where such non-linear terms appear (e.g. from (12), $P(q_A, q_{A2}) = p_1\pi_1 + p_2\pi_2 q_A + p_2\pi_2 q_{A2} - p_2\pi_2 q_A q_{A2}$ has the nonlinear term $p_2\pi_2 q_A q_{A2}$). Specifically, from Theorem 2 above, if $(q_1, q_2, \cdots, q_N)$ is an optimal foraging behavior, it is a NE of the $N-$player agent-normal form of the optimal foraging game.

For the converse, assume that $(x, y, z) \in \Delta^1 \times \Delta^2 \times \Delta^3$ is a NE of a three-player agent-normal form of the optimal foraging game. Then

$$\frac{P(x', y, z)}{Q(x', y, z)} \leq \frac{P(x, y, z)}{Q(x, y, z)}, \frac{P(x, y', z)}{Q(x, y', z)} \leq \frac{P(x, y, z)}{Q(x, y, z)}, \text{ and } \frac{P(x, y, z')}{Q(x, y, z')} \leq \frac{P(x, y, z)}{Q(x, y, z)}$$

for all $(x', y', z') \in \Delta^1 \times \Delta^2 \times \Delta^3$. Thus

$$\begin{aligned} P(x', y, z)Q(x, y, z) &\leq P(x, y, z)Q(x', y, z), \\ P(x, y', z)Q(x, y, z) &\leq P(x, y, z)Q(x, y', z), \\ P(x, y, z')Q(x, y, z) &\leq P(x, y, z)Q(x, y, z'). \end{aligned}$$

Adding these three inequalities yields

$$\begin{aligned} &(P(x', y, z) + P(x, y', z) + P(x, y, z'))\, Q(x, y, z) \\ &\leq P(x, y, z)\,(Q(x', y, z) + Q(x, y', z) + Q(x, y, z'))\,, \end{aligned}$$

Since P is linear, $P(x', y, z) + P(x, y', z) + P(x, y, z') = P(x', y', z') + 2P(x, y, z)$. Similarly $Q(x', y, z) + Q(x, y', z) + Q(x, y, z') = Q(x', y', z') + 2Q(x, y, z)$. Thus, (1) implies that $P(x', y', z')Q(x, y, z) \leq P(x, y, z)Q(x', y', z')$. That is, $\frac{P(x',y',z')}{Q(x',y',z')} \leq \frac{P(x,y,z)}{Q(x,y,z)}$ for $(x', y', z') \in \Delta^1 \times \Delta^2 \times \Delta^3$. That is, (x, y, z) yields optimal foraging behavior.

The final statement of the theorem is obvious.

■

Foraging with simultaneous resource encounters.

Nash equilibrium strategy (q_{AB}, q_B).

The three-player agent normal form of Figure 3 has strategy set $\Delta^1 \times \Delta^2 \times \Delta^3$ where $\Delta^1 = \{(q_A, 1 - q_A) \mid 0 \leq q_A \leq 1\})$, $\Delta^2 = \{(q_{AB}, q_{BA}, 1 - q_{AB} - q_{BA}) \mid 0 \leq q_{AB} \leq 1, 0 \leq q_{BA} \leq 1 - q_{AB}\})$ and $\Delta^3 = \{(q_B, 1 - q_B) \mid 0 \leq q_B \leq 1\})$.

From (6), $\tau^2 \frac{\partial f}{\partial q_A}$ and $\tau^2 \frac{\partial f}{\partial q_{AB}}$ are equal to

$$p_{AA} \left[\pi_A \tau_s + (p_{AB} q_{BA} + p_{BB} q_B)(\pi_A \tau_{Bh} - \pi_B \tau_{Ah})\right] > 0.$$

and

$$p_{AB} \left[\pi_A \tau_s + (p_{AB} q_{BA} + p_{BB} q_B)(\pi_A \tau_{Bh} - \pi_B \tau_{Ah})\right] > 0.$$

respectively. In particular, both of these partial derivatives are positive since prey A is the most profitable.

From the first displayed inequality, $f(1, q_{AB}, q_{BA}, q_B) > f(q_A, q_{AB}, q_{BA}, q_B)$ for all $0 \leq q_A < 1$. That is, $q_A = 1$ strictly dominates all other actions of player 1 and so, at a NE, resource A must be consumed whenever encountered on its own.

From the second inequality, $f(q_A, 1-q_{BA}, q_{BA}, q_B) > f(q_A, q_{AB}, q_{BA}, q_B)$ for all $0 \leq q_{AB} < 1 - q_{BA}$. Thus, a strategy of player 2 whereby a resource is not always consumed at its decision node AB (i.e. $q_{AB} + q_{BA} < 1$) is strictly dominated by the strategy $(1 - q_{BA}, q_{BA}, 0)$. It follows from this that $q_{AB} + q_{BA} = 1$ at any NE.

That is, optimal foraging emerges from the reduced tree of Figure 3. This is the two-player agent normal form game with strategy set $\{(q_{AB}, 1 - q_{AB}) \mid 0 \leq q_{AB} \leq 1\} \times \{(q_B, 1 - q_B) \mid 0 \leq q_B \leq 1\}$ and common payoff function (7) given by

$$f(q_{AB}, q_B) = \frac{(p_A + p_{AB} q_{AB})\,\pi_A + (p_{AB}(1 - q_{AB}) + p_B q_B)\,\pi_B}{\tau}$$

where

$$\tau = \tau_s + p_A \tau_{Ah} + p_{AB} q_{AB} \tau_{Ah} + p_{AB}(1 - q_{AB})\tau_{Bh} + p_B q_B \tau_{Bh}.$$

Thus, $\tau^2 \frac{\partial f}{\partial q_{AB}}$ is equal to

$$p_{AB}\left[(\pi_A - \pi_B)\,\tau_s + (p_A + p_{AB} + p_B q_B)\,(\pi_A \tau_{Bh} - \pi_B \tau_{Ah})\right]$$

and this is positive if and only if

$$q_B > q_B^* = \frac{(\pi_B - \pi_A)\tau_s - (p_A + p_{AB})(\pi_A \tau_{Bh} - \pi_B \tau_{Ah})}{p_B(\pi_A \tau_{Bh} - \pi_B \tau_{Ah})}.$$

That is, the best response to player 2 is given by (8). Similarly, $\tau^2 \frac{\partial f}{\partial q_B}$ is equal to

$$p_B\left[\pi_B \tau_s + (p_A - p_{AB} q_{AB})\,(\pi_A \tau_{Bh} - \pi_B \tau_{Ah})\right]$$

and this is negative if and only if

$$q_{AB} > q_{AB}^* = \frac{\pi_B \tau_s + p_A(\pi_A \tau_{Bh} - \pi_B \tau_{Ah})}{p_{AB}(\pi_A \tau_{Bh} - \pi_B \tau_{Ah})}.$$

That is, the best response to player 1 is given by (10).

The optimal strategy (q_{AB}, q_B) as a function of energy value π_B of the less profitable prey type.

When prey A handling time is shorter than prey B handling time (i.e. $\tau_{Ah} < \tau_{Bh}$), $\frac{\pi_A \tau_{Bh}}{\tau_{Ah}} > \pi_{B1}^* > \pi_{B2}^* > \pi_{B3}^* > \pi_{B4}^*$.

For $0 < \pi_B < \pi_{B4}^*$, $q_{AB}^* < 0$ and $q_B^* < 0$ and the optimal strategy is $(q_{AB}, q_B) = (1, 0)$ (Figure 4a). For $\pi_{B4}^* < \pi_B < \pi_{B3}^*$, $0 < q_{AB}^* < 1$ and $q_B^* < 0$ and the optimal strategy is $(q_{AB}, q_B) = (1, 0)$ (Figure 4b).

For $\pi^*_{B3} < \pi_B < \pi^*_{B2}$, $1 < q^*_{AB}$ and $q^*_B < 0$ and the optimal strategy is $(q_{AB}, q_B) = (1, 1)$ (Figure 4c). For $\pi^*_{B2} < \pi_B < \pi^*_{B1}$, $1 < q^*_{AB}$ and $0 < q^*_B < 1$ and the optimal strategy is $(q_{AB}, q_B) = (1, 1)$ (Figure 4d). For $\pi^*_{B1} < \pi_B < \pi_A \tau_{Bh}/\tau_{Ah}$, $1 < q^*_{AB}$ and $1 < q^*_B$ and the optimal strategy is $(q_{AB}, q_B) = (0, 1)$ (Figure 4e).

The Nash equilibria of the prey recognition game

From the main text, the energy intake rate in the prey recognition game of Figure 6 is

$$f((q_A, q_R, q_M), q_{A1}, q_{A2}) = \frac{p_1\pi_1(q_A + q_R q_{A1}) + p_2\pi_2(q_A + q_R q_{A2})}{\tau}$$

where $\tau = \tau_s + p_1 q_A \tau_{1h} + p_1 q_R(q_{A1}\tau_{1h} + \tau_r) + p_2 q_A \tau_{2h} + p_2 q_R(q_{A2}\tau_{2h} + \tau_r)$. It is straightforward to show that $f((\lambda q_A, 1-\lambda, \lambda q_R), q_{A1}, q_{A2}) < f((q_A, 0, q_R), q_{A1}, q_{A2})$ for $0 \le \lambda < 1$ if $q_A + q_R = 1$. Thus, any strategy $(\lambda q_A, 1-\lambda, \lambda q_R)$ of player 1 with $0 \le \lambda < 1$ is strictly dominated by $(q_A, 0, q_R)$ and so $q_M = 0$ at any NE.

Furthermore

$$\tau^2 \frac{\partial f((q_A, q_M, q_R), q_{A1}, q_{A2})}{\partial q_{A1}} = p_1 q_R(\pi_1(p_1 q_R \tau_r + \tau_s + p_1\tau_s) + p_2(q_A + q_{A2}q_R)(\pi_1\tau_{2h} - \pi_2\tau_{1h}) + p_2 q_A \pi_1 \tau_s + p_2 q_R \pi_1(\tau_r + \tau_s))$$

is positive if $q_R > 0$ since $\frac{\pi_1}{\tau_{1h}} > \frac{\pi_2}{\tau_{2h}}$. That is, at a NE, the predator never moves immediately to another patch if it encounters a prey (i.e. $q_M = 0$) and, if the predator does spend some recognition time (i.e. $q_R > 0$), then it must attack any prey 1 that it recognizes (i.e. $q_{A1} = 1$).

As in the main text, we now look for the NE of the two-player game corresponding to the reduced tree of Figure 6. Here,

$$f(q_A, q_{A2}) = \frac{p_1\pi_1 + p_2\pi_2(q_A + (1-q_A)q_{A2})}{\tau}$$

where $\tau = \tau_s + p_1\tau_{1h} + p_2\tau_{2h}(q_A + (1-q_A)q_{A2}) + (p_1 + p_2)\tau_r(1 - q_A)$. For the best response of player 1 to strategy q_{A2} of player 2, we calculate the derivative of f with respect to q_A as

$$\frac{p_2(1-q_{A2})(p_1\pi_2\tau_{1h} - p_1\pi_1\tau_{2h} + \pi_2\tau_s) + \tau_r(p_1+p_2)(p_1\pi_1 + p_2\pi_2)}{\tau^2}.$$

Since $\tau_r > 0$, $\frac{\partial f}{\partial q_A} > 0$ if $\pi_2/\tau_{2h} \ge p_1\pi_1/(p_1\tau_{1h} + \tau_s)$ and so any NE then has $q_A = 1$. Furthermore, in this case, $f(1, q_{A2}) = (p_1\pi_1 + p_2\pi_2)/(p_1\tau_{1h} + p_2\tau_{2h} + \tau_s)$ does not depend on q_{A2}. Thus, every strategy pair of the form $(1, q_{A2})$ with $0 \le q_{A2} \le 1$ is a NE.

For the remainder of this Appendix, assume

$$\pi_2/\tau_{2h} < p_1\pi_1/(p_1\tau_{1h} + \tau_s). \tag{S2}$$

Then derivative of f with respect to q_A is positive provided $q_{A2} > q^*_{A2}$, as in (13). That is, the best response of player 1 to the strategy q_{A2} of player 2 is given by (13).

Similarly, the derivative of f with respect to q_{A2} is

$$\frac{p_2(q_A - 1)(p_2(q_A - 1)\pi_2\tau_r + p_1(\pi_1\tau_{2h} - \pi_2(\tau_{1h} + \tau_r - q_A\tau_r)) - \pi_2\tau_s)}{\tau^2}.$$

This derivative equals 0 when either $q_A = 1$, or

$$q_A = q^*_A = 1 - \frac{p_1\pi_1\tau_{2h} - \pi_2\,(\tau_s + p_1\tau_{1h})}{\pi_2(p_1 + p_2)\tau_r}.$$

Under (S2), $1 > q^*_A$ and for $q^*_A < q_A < 1$ (respectively, $0 \le q_A < q^*_A$), the best response of player 2 is $q_{A2} = 0$ (respectively, $q_{A2} = 1$) because the derivative is negative (respectively, positive). Finally, when $q_A = 1$ or $q_A = q^*_A$, the derivative of f equals zero and so q_{A2} can be any value between 0 and 1. This gives (14).

Literature cited

1. Johns MV Jr, Miller RG Jr (1963) Average renewal loss rates. The Annals of Mathematical Statistics, 34,2: 396-401.

2. Stephens DW, Krebs JR (1986) Foraging theory. Princeton, NJ: Princeton University Press.

3. Houston AI, McNamara JM (1999) Models of adaptive behaviour. Cambridge, UK: Cambridge University Press.

4. Charnov EL, Orians GH (1973) Optimal foraging: Some theoretical explorations. http://hdl.handle.net/1928/1649.

5. Garay J, Mori TF (2010) When is the opportunism remunerative? Community Ecology 11: 160-170.

6. Wald A (1944) On cumulative sums of random variables. The Annals of Mathematical Statistics 15: 283-296.

7. Cressman R (2003) Evolutionary Dynamics and Extensive Form Games. Cambridge, MA: The MIT Press.
